# Profiling of terminating ribosomes reveals translational control at stop codons

**DOI:** 10.1101/2025.09.16.676599

**Published:** 2025-09-18

**Authors:** Longfei Jia, Yuanhui Mao, Saori Uematsu, Xinyi Ashley Liu, Leiming Dong, Leonardo Henrique França de Lima, Shu-Bing Qian

**Affiliations:** 1 Division of Nutritional Sciences, Cornell University, Ithaca, NY 14853, USA.; 2 Laboratory of Molecular Modelling and Bioinformatics (LAMMB), Department of Physical and Biological Sciences, Campus Sete Lagoas, Universidade Federal de São João Del Rei, Sete Lagoas, Brazil.; 3 Present address: State Key Laboratory of Reproductive Medicine and Offspring Health, Nanjing Medical University, Nanjing, P.R.China; 4 Present address: Liangzhu Laboratory, Zhejiang University, Hangzhou, P.R.China; 5 These authors contributed equally; 6 Lead contact

## Abstract

Accurate termination of protein synthesis is paramount for the integrity of cellular proteome, but our understanding of the dynamics and fidelity of terminating ribosomes is far from complete. Here we establish profiling of terminating ribosomes in mammalian cells and report a wide range of ribosome pausing at individual stop codons. We identify a sequence motif upstream of the stop codon that contributes to termination pausing, which was confirmed by massively paralleled reporter assays. Unexpectedly, lack of termination pausing increases the chance of stop codon slippage, generating proteins with mixed C-terminal extensions. We demonstrate that the sequence-dependent termination pausing is a result of post-decoding mRNA scanning by the 3’ end of 18S rRNA. We further observe tissue-specific termination pausing that correlates with the stoichiometry of Rps26, which constrains mRNA:rRNA interaction. Thus, termination pausing represents a translational signature associated with mRNA sequence contexts, ribosome heterogeneity, and cell type-specific translational control.

## INTRODUCTION

Eukaryotic mRNA translation concludes when a stop codon (UAA, UAG, and UGA) enters the A site of the ribosome followed by concerted actions of release factors eRF1 and eRF3 ^1^. Mimicking the tRNA molecule, eRF1 is responsible for stop codon recognition and cleavage of peptidyl-tRNA bond, while eRF3 facilitates this process in a GTP-dependent manner ^2^. Alongside these release factors, the ribosome recycling factor ABCE1 contributes to the completion of translation termination ^3,4^. Upon eRF1-mediated release of the nascent polypeptide chain from the P-site, ABCE1 promotes the splitting of the 80S ribosome into 60S and 40S subunits. The subsequent dissociation of the deacylated tRNA and mRNA from the liberated 40S subunit ensures complete recycling of ribosomes and mRNAs.

Although translation termination is a highly efficient process, the three stop codons have varied efficiency on individual mRNAs ^5^. Notably, the least efficient stop codon UGA is the most frequent one in human transcriptome. When stop codons are decoded as sense codons by near-cognate tRNAs, the subsequent stop codon readthrough (SCR) gives rise to protein products with C-terminal extension ^6^. It has been well-documented that the sequence context surrounding the stop codon influences the fidelity of translation termination ^7,8^. For instance, the presence of a C following the stop codon (+4 position) increases the likelihood of SCR. This is consistent with the notion that eRF1 binding allows accommodation of four nucleotides in the A site ^9^. Additionally, formation of RNA secondary structures downstream of the stop codon stimulates readthrough, as exemplified by the *kelch* mRNA in *Drosophila* ^10^. It has been proposed that downstream stem loops lead to ribosomes pausing at a stop codon, thereby nudging competition in favor of aminoacyl-tRNA decoding. Ribosome stalling at stop codons could also trigger frameshifting as exemplified by OAZ translation ^11^. Unlike SCR that follows the same reading frame after the stop codon, termination-coupled frameshifting gives rise to protein products with varied C-terminal extensions. Such promiscuous translation in 3’UTR has been documented in cells lacking ABCE1 ^12^. Additionally, terminating ribosomes could undergo reinitiation via bidirectional migration depending on the sequence context ^13^. The diverse behavior of terminating ribosomes strongly suggests that ribosome dynamics at stop codons is coupled with termination fidelity. However, very little is known whether individual stop codons undergo differential termination kinetics, and if so, what is the underlying mechanism.

Ribosome profiling allows for global examination of translation termination for all stop codons in their native sequence contexts ^14^. Application of this approach to a wide range of cell types reveals that translational readthrough in cellular mRNAs may be more widespread than previously appreciated ^15^. Interestingly, there is an exquisite tissue specificity with readthrough occurring at elevated levels in the central nervous system ^16,17^. By contrast, the reproductive system like testis tends to have minimal readthrough events ^10^. These findings suggest that translational readthrough is regulated not only by the sequence context, but also by *trans*-factors acting on terminating ribosomes. The mechanistic basis of cell type-specific termination fidelity remains largely unexplored.

Ribosome stalling at premature termination codons has been associated with nonsense-mediated mRNA decay ^18^. However, this notion has been challenged by recent findings that premature and normal termination codons exhibit similar ribosome occupancy ^19^. As nonsense mutations account for >10% of inherited human diseases, such as cystic fibrosis and muscular dystrophy ^20^, it is highly desirable to induce readthrough at premature termination codons without disrupting termination at normal stop codons. A better understanding of ribosome dynamics at individual stop codons is important to identify molecular events that could be targeted by nonsense-suppressive therapeutics.

By profiling terminating ribosomes in mammalian cells and mouse tissues, here we report a broad range of termination dynamics across the entire transcriptome. The dwell time of terminating ribosomes is not only influenced by the sequence context, but also subjected to regulation by ribosome composition. Unexpectedly, ribosome pausing at stop codons acts as a translational control by preventing ribosome sliding and downstream 3’UTR translation. We define the underlying mechanism by uncovering sequence-dependent rRNA:mRNA interaction, highlighting the physiological significance of stop codon-associated translational control.

## RESULTS

### High-resolution Ribo-seq reveals dynamic features of terminating ribosomes

**Fig. 1.**
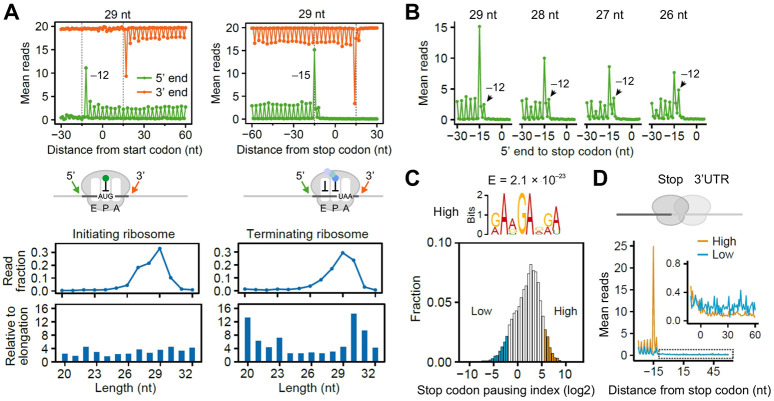
Characterizing terminating ribosomes using Ribo-seq. **(A)** Aggregation plots of mean Ribo-seq reads around the start (left) and stop (right) codons. Both 5’ end (green) and 3’ end (orange) of footprint reads (29 nt) were used for mapping. For bottom panels, the line plots show the distribution of reads with different lengths, whereas the bar plots show the read distribution of initiating (left) and terminating (right) ribosomes relative to elongation ribosomes. **(B)** Aggregation plots of mean Ribo-seq reads around the stop codon. The reads were stratified by the length followed by mapping using the 5’ end of footprint reads. **(C)** A histogram plot of ribosome pausing index at individual stop codon. The top shows the enriched sequence motif of mRNAs with strong ribosome pausing at stop codons. **(D)** Aggregation plots of mean Ribo-seq reads around the stop codon. “High” and “Low” refer to mRNAs with differential pausing indexes shown in (C). The 3’UTR read density was highlighted in the insert.

Ribosome profiling offers a snapshot of global translation inside cells by sequencing ribosome-protected mRNA fragments (RPFs) ^21^. Relative to the coding sequence (CDS), ribosome footprints tend to accumulate at start and stop codons, respectively. We recently developed Ezra-seq that enables high-resolution ribosome profiling with excellent 5’ end accuracy of footprints ^22^. With superior 3-nt periodicity, Ezra-seq enables detection of start codon-associated ribosome frameshifting ^22^. We reasoned that Ezra-seq would allow us to assess the behavior of terminating ribosomes as well. Indeed, we observed clear boundaries of terminating ribosomes when mRNAs are aligned to their annotated stop codons (Figure 1A, right panel). Unlike initiating ribosomes that have the AUG codon at the P site, terminating ribosomes have their stop codons at the A site as evidenced by the prominent 5’ end peak at −15 nt. Consistent with previous reports ^14^, terminating ribosomes protect additional nucleotides (Figure 1A, bottom panel). Relative to elongating ribosomes, terminating ribosomes exhibit two read populations with long (30 – 31 nt) and short (20 – 23 nt) footprints, which was not seen for initiating ribosomes (Figure 1A, bottom panel). These short footprints likely represent terminating ribosomes with an empty A-site.

A close inspection of stop codon footprints revealed an additional peak at −12 nt, which becomes more prominent when the reads are shorter (Figure 1B). Previous toe-printing assays reported a forward movement of terminating ribosomes in the presence of eRF1, resulting in the leading edge shifted from +13 nt to +15 nt ^23^. Recent single molecule study of translation termination uncovered pre- and post-termination phases catalyzed by eRF1 ^24^. It is possible that the two distinct 5’ end peaks represent pre- and post-terminating ribosomes with the latter assuming the rotated conformation. We could not rule out the possibility that these terminating ribosomes have the stop codons at the P-site prior to disassembly.

To assess the kinetics of terminating ribosomes, we conducted cycloheximide (CHX) chase in HEK293 cells. CHX treatment stalls the elongating ribosomes on the messenger but not the terminating ribosomes ^25^. While 5 min CHX treatment markedly reduced the footprint density at stop codons, 30 min pre-treatment nearly abolished the stop codon peaks (Figure S1A and S1B). Therefore, terminating ribosomes undergo pausing but not stalling at the stop codon. Supporting this notion, we barely observed stacked trailing ribosomes upstream of the stop codon, a sign indicative of ribosome collision.

Despite the prominent termination pausing, the ribosome density at individual stop codons varies a lot with several orders of magnitude. We computed stop codon pausing index on individual mRNAs by normalizing the read density at the stop codon with the averaged CDS occupancy to factor out differential mRNA abundance and translation efficiency (Figure 1C). To search for sequence elements controlling termination pausing, we compared mRNAs with high and low pausing index. While no specific sequences were found after the stop codon, an upstream GA-sequence motif was enriched on mRNAs with strong termination pausing (*E* = 2.1 × 10^−23^, Figure 1C). Therefore, certain coding sequences upstream of the stop codon influence the dynamics of terminating ribosomes.

For mRNAs without termination pausing, it is possible that the 80S ribosome either rapidly dissociates from the messenger or undergoes stop codon readthrough. These two scenarios are expected to result in different ribosome density in 3’UTR. Intriguingly, we observed higher 3’UTR read density on mRNAs lacking termination pausing (Figure 1D). This was not due to biased downstream sequences as the +4 nucleotide minimally affected the 3’UTR translation (Figure S1C). Notably, the 3’UTR reads exhibited poor 3nt periodicity despite the superior phasing of the CDS reads (Figure S1D). Therefore, stop codon readthrough could not fully explain the elevated 3’UTR read density on mRNAs lacking stop codon peaks.

### Profiling of terminating ribosomes by eRF1-seq

**Fig. 2.**
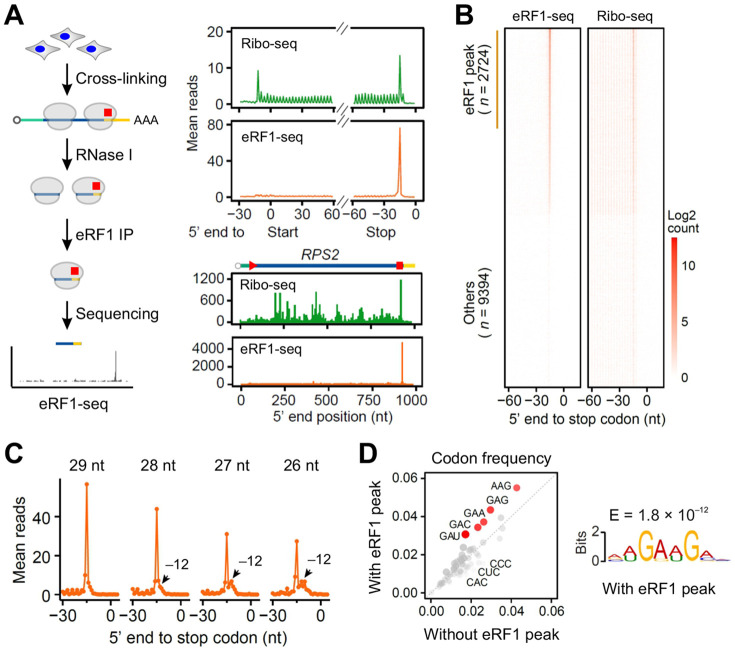
Characterizing terminating ribosomes using eRF1-seq. **(A)** The left panel shows the schematic of eRF1-seq procedures. The right top panel shows the aggregation plots of Ribo-seq (green) and eRF1-seq (orange). The right bottom panel shows a representative mRNA (*RPS2*) with reads obtained from Ribo-seq and eRF1-seq. The 5’ end of reads were used for mapping. **(B)** Heatmaps of individual mRNAs with reads obtained from Ribo-seq (right) and eRF1-seq (left). **(C)** Aggregation plots of mean eRF1-seq reads around the stop codon. The reads were stratified by the length followed by mapping using the 5’ end of footprint reads. **(D)** Comparison of codon frequencies upstream of stop codons between mRNAs with and without eRF1 peaks. The right panel shows the enriched sequence motif for mRNAs with eRF1 peaks at stop codons.

To better assess the dynamics of translation termination, we developed terminating ribosome profiling by collecting ribosomes associated with eRF1 (Figure 2A). HEK293 cells were first crosslinked by formaldehyde followed by cell lysis and RNase I digestion. From the monosome separated by sucrose gradient, eRF1-bound ribosomes were enriched by immunoprecipitation as confirmed by Western blotting (Figure S1E). Without crosslinking, ribosomal proteins were minimally pulled down by the eRF1 antibody, confirming the transient nature of eRF1 binding. The subsequent Ribo-seq revealed a marked accumulation of reads at the annotated stop codons (Figure 2A, right panel). Remarkably, eRF1-seq maintained the single nucleotide resolution, permitting unambiguous identification of termination sites on the endogenous mRNAs (Figure 2B). Like Ribo-seq, we also observed a forward shifting of post-terminating ribosomes from eRF1-seq (Figure 2C). With excellent reproducibility between biological replicates (Figure S1F), eRF1-seq effectively captures the footprints of terminating ribosomes.

In agreement with the Ribo-seq data sets, eRF1-seq revealed that not all the mRNAs exhibited eRF1 peaks at the annotated stop codons (Figure 2B), echoing the wide range of termination pausing. By comparing mRNAs with or without eRF1 peaks, we found that neither the 3’UTR length nor the sequence features after the stop codon contribute to the varied eRF1 peak density (Figure S1G). However, sequences preceding the stop codon showed an enrichment of GA-sequence motif on mRNAs with prominent eRF1 peaks (Figure 2D). This result is consistent with the Ribo-seq analysis (Figure 1C), confirming that termination pausing is an inherent feature of individual mRNAs. Notably, three different stop codons show similar pausing features and sequence motifs (Figure S1G and S1I).

### eRF1-seq reveals alternative termination sites

**Fig. 3.**
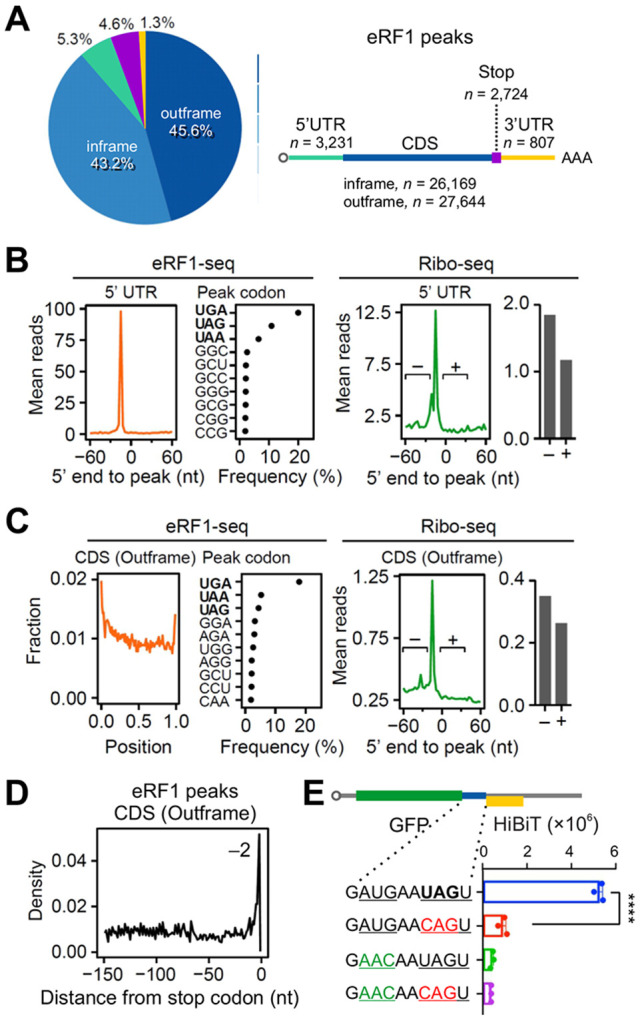
eRF1-seq reveals prevailing termination sites. **(A)** A pie chart shows fractions of eRF1 peaks mapped to different mRNA regions. “Inframe” and “Outframe” refer to the positions of eRF1 peaks within CDS relative to the annotated start codons. **(B)** The left panel shows mean eRF1-seq reads around the position of eRF1 peaks within 5’ UTR. The dot plot shows the frequency of A-site codons at eRF1 peaks. The right panel shows mean Ribo-seq reads around the position of eRF1 peaks within 5’ UTR. The bar graph shows the mean ribosome densities before (−) and after (+) the eRF1 peaks. **(C)** The left panel shows mean eRF1-seq reads around the position of out-of-frame eRF1 peaks within CDS. The dot plot shows the frequency of A-site codons at eRF1 peaks. The right panel shows mean Ribo-seq reads around the position of out-of-frame eRF1 peaks within CDS. The bar graph shows the mean ribosome densities before (−) and after (+) the eRF1 peaks. **(D)** The distribution of eRF1 peaks before the annotated stop codon. Only the out-of-frame eRF1 peaks were used for plotting. **(E)** A bar graph shows the HiBiT-based reporter assays in HEK293-K^b^ cells. HiBiT signals were measured from cells transfected with mRNA reporters bearing different sequences between GFP and HiBiT. Error bars, mean ± s.e.m. *n* = 3 biological replicates. ****P ≤ 0.0001 by unpaired two-tailed *t*-test.

Despite the prominent peak at the annotated stop codons, individual eRF1 peaks were broadly distributed from 5’UTR to 3’UTR (Figure 3A). The presence of eRF1 peaks in 5’UTR is not entirely surprising because ~50% of human mRNAs contain upstream open reading frames (uORFs) ^26^. Indeed, ~30% of eRF1 positions in 5’UTR corresponds to the termination sites of uORFs previously identified by Ribo-seq (Figure S2A). By revealing additional termination sites in 5’UTR that were previously uncharacterized, eRF1-seq may prove to be a substantial aid in expanding the scope of uORFs. Nearly all the A-site codons of eRF1 peaks in 5’UTR are typical stop codons (Figure 3B). Additionally, Ribo-seq showed lowered read density after those sites (Figure 3B, right panel), a sign of true termination. Besides 5’UTR, we also identified in 3’UTR a total of 807 eRF1 peaks, which shared similar features as the peaks in 5’UTR (Figure S2B). Compared to Ribo-seq that typically shows low amount of reads in 3’UTR, eRF1-seq confers a higher signal/noise ratio in assessing 3’UTR translation.

Even with stringent peak calling, eRF1-seq revealed substantial number of peaks in the coding region. Many eRF1 peaks in CDS have their A sites positioned at in-frame sense codons (Figure S2C). Notably, the majority of those codons are shifted stop codons. For instance, CUG is a −1 frameshifted UGA, whereas GAG, GAA, and AAG are +1 frameshifted UGA or UAA. Despite the presence of eRF1 peaks, those sense codons do not signal translation termination because Ribo-seq showed no reduction of read density downstream (Figure S2C). Given the evident ribosome pausing at those “stop-like” codons, it appears that eRF1 competes with A-site tRNAs during elongation, resulting in false termination.

The eRF1 peaks in CDS are not limited to in-frame sense codons. A large number of out-of-frame eRF1 peaks correspond to typical stop codons especially UGA (Figure 3C). Supporting true translation termination, Ribo-seq showed lowered read density after those out-of-frame termination sites (Figure 3C, right panel). Interestingly, most out-of-frame termination events occur in the beginning of CDS. Besides overlapping uORFs, this result further supports start codon-associated ribosome frameshifting we reported recently ^22^. Additionally, out-of-frame eRF1 peaks are also enriched near the end of CDS (Figure 3C and 3D), likely due to migration of terminating ribosomes at stop codons to search for upstream start codons (Figure 3D). We confirmed the stop codon-associated reinitiation by placing between GFP and HiBiT a 9 nt sequence derived from *CASQ2*, which contains an out-of-frame AUG codon upstream of the stop codon UAG (Figure 3E). Consistent with previous reports ^27^, mutating the stop codon UAG abolished the reinitiation event that drives out-of-frame HiBiT translation (Figure 3E).

### Sequence determinants of termination pausing

**Fig. 4.**
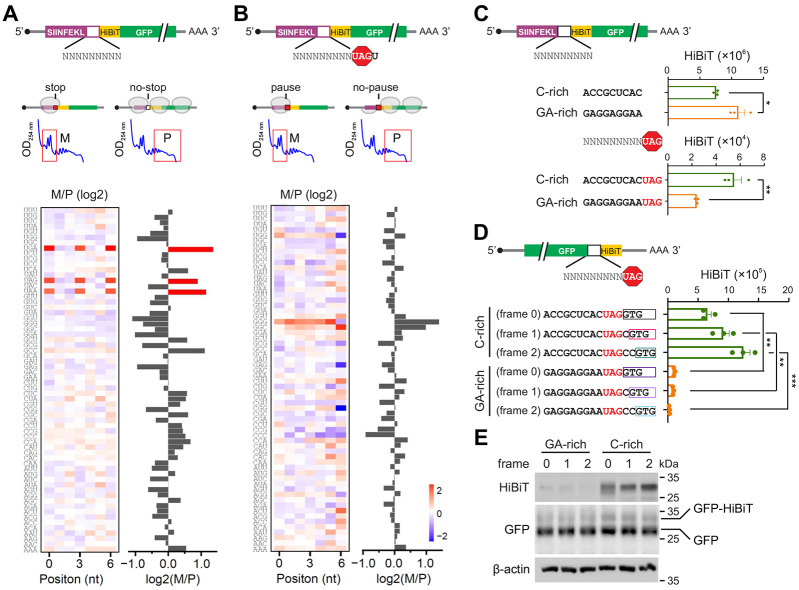
Termination pausing is influenced by sequence contexts. **(A)** The up panel shows the schematic of a massively parallel reporter assay, where the stop codon of uORF was replaced with a random 9-nt sequences. The uORF translation was monitored by the number of associated ribosomes separated by sucrose gradient (M, monosome; P, polysome). The heatmap shows the ratio of monosome fraction over polysome fraction (M/P) when different codons were placed at individual positions within the 9-nt random sequence (left). The stop codons UGA, UAG and UAA are highlighted. The bar graph (right) shows the mean M/P ratio averaged across all positions. **(B)** Similar as (A) except that the random 9-nt sequences were placed before the stop codon UAG. **(C)** Bar graphs show the HiBiT signals in HEK293-K^b^ cells transfected with mRNA reporters bearing C-rich or GA-rich sequences between the uORF and HiBiT-GFP. The up panel shows the control without the stop codon UAG. Error bars, mean ± s.e.m. *n* = 3 biological replicates. *P ≤ 0.05; **P ≤ 0.01 by unpaired two-tailed *t*-test. **(D)** A bar graph shows the HiBiT signals in HEK293-K^b^ cells transfected with mRNA reporters bearing C-rich or GA-rich sequence before the GFP stop codon UAG. The downstream HiBiT sequence was inserted into different reading frames. Error bars, mean ± s.e.m. *n* = 3 biological replicates. **P ≤ 0.01; ***P ≤ 0.001 by unpaired two-tailed *t*-test. **(E)** Representative Western blots of GFP and HiBiT in HEK293-K^b^ cells transfected with C-rich or GA-rich reporters as described in (D).

Given the wide range of termination pausing at individual stop codons, we next explored the sequence determinants of termination pausing using a massively paralleled reporter assay (MPRA). Unlike analyzing endogenous genes with sequences shaped by evolution, MPRA relies on completely randomized sequences to identify all possible sequence elements in an unbiased manner. We previously employed a uORF reporter to evaluate start codon usage within randomized sequences ^28^. In this system, mRNA variants with efficient translation of uORF reside in monosome, whereas downstream GFP translation relocates the messengers to polysome. A monosome/polysome ratio would infer the uORF translation. To probe the stop codon usage, we modified the uORF reporter by replacing the stop codon of uORF with a 9-nt long random sequence (Figure 4A). Appearance of an in-frame stop codon within the insert would exclusively place the mRNA in monosome. We synthesized the mRNA library using *in vitro* transcription to avoid plasmid-based transcriptional variation. In HEK293 cells transfected with the mRNA pool, we separated monosome and polysome using sucrose gradient followed by deep sequencing. For mRNA variants recovered from monosome, we observed an enrichment of all three stop codons (UGA, UAG, UAA) (Figure 4A). Importantly, these stop codons showed prominent in-frame positions within the insert (Figure S3A and S3B). Codons appeared in other reading frames are also meaningful. For instance, codons enriched in frame 2 belong to NUA and NUG, another indication of in-frame stop codons (Figure S3B, bottom panel). Therefore, MPRA is suitable for dissecting the sequence context of stop codons.

Ribosomal pausing at uORF stop codons is expected to retain the mRNA in monosome by preventing leaky scanning and stop codon readthrough. This feature allows us to evaluate the sequence determinants of termination pausing based on the monosome/polysome ratio. We first placed the 9-nt long random sequence immediately after the stop codon UAG (Figure S3C). Despite the prior finding that a C at the +4 nt position promoted readthrough ^7^, we found that mRNA variants bearing the C-rich sequence after the stop codon were depleted from both monosome and polysome fractions. This is likely due to the faster turnover of these mRNAs because of 3’UTR translation ^29,30^. Consistent with Ribo-seq and eRF1-seq, no specific sequences after UAG were over-represented in monosome (Figure S3D), suggesting that sequences after the stop codon have limited effect on the dynamics of terminating ribosomes.

To assess whether the upstream sequences influence the stop codon fidelity, we inserted the 9-nt long random sequence before the stop codon UAG (Figure 4B). Among all the mRNA variants, a G-rich sequence was evidently enriched in monosome (Figure 4B and S3E). Notably, the G-rich sequence motif showed no reading frame information, suggesting that it is the sequence rather than the codon identity that contributes to ribosome pausing at stop codons. Interestingly, a C-rich sequence was relatively depleted from the monosome (Figure 4B), suggesting that the C-rich sequence preceding the stop codon promotes readthrough and downstream translation. These results are in line with the sequence specificity in termination pausing revealed by Ribo-seq and eRF1-seq.

### Stop codon-associated random translation

It is not immediately clear whether the 3’UTR translation is a result of stop codon readthrough or stop codon-associated reinitiation. To probe the nature of downstream translation triggered by upstream C-rich sequences, we constructed individual mRNA reporters by placing a C-rich sequence before the uORF stop codon UAG (Figure 4C). Since many C-rich triplets encode proline, we avoided this imino acid by choosing non-proline C-rich codons. As a control, we used a GA-sequence selected from the eRF1-seq. To detect 3’UTR translation, we introduced immediately after the stop codon a sequence encoding HiBiT that can be detected with superior sensitivity ^28^. The start codon of HiBiT was omitted to exclude leaky scanning-mediated translation. In the absence of the UAG stop codon, the GA-rich sequence produced higher HiBiT signals than the C-rich sequence, presumably due to the different amino acids encoded by the insert. In the presence of the stop codon, however, the C-rich sequence nearly doubled HiBiT signals compared to the GA-rich sequence (Figure 4C, bottom panel). This result is congruent with the MPRA assay, suggesting that the C-rich coding sequence preceding the stop codon not only reduces termination pausing, but also promotes downstream translation. This feature is not limited to uORF because we obtained similar results when the uORF was replaced by the GFP coding sequences (Figure S3F).

Stop codon readthrough is expected to follow the in-frame translation and generate a fusion protein with C-terminal extension. To test this possibility, we placed the HiBiT sequence into different reading frames of the stop codon. To our surprise, we observed even higher HiBiT signals from both frame 1 and frame 2 reporters (Figure 4D). By contrast, the GA-sequence preceding the stop codon largely suppressed the downstream HiBiT translation. We observed the same feature when different sequences were used (Figure S3G). To directly examine the translational products, we conducted immunoblotting of whole cell lysates using HiBiT antibodies (Figure 4E). GFP-fusion proteins (~ 30 kDa) were readily detected in C-rich reporters regardless of the HiBiT reading frames, ruling out the possibility of reinitiation. Notably, the non-fusion GFP showed comparable levels, arguing that the differential HiBiT translation was neither due to varied initiation nor the altered amino acids encoded by the insert. Therefore, the C-rich coding sequence triggers ribosome sliding at the stop codon, resulting in 3’UTR translation in all three reading frames.

We next examined the positional effects of the C-rich sequence by introducing the C triplet into different positions upstream of the stop codon. The HiBiT reporter assay showed that the C triplet immediately preceding the stop codon was the least potent in triggering the HiBiT translation in 3’UTR (Figure S3H). This result suggests that the sequence specificity lies at the upstream of the E site.

### ABCE1 regulates terminating ribosomes independent of the sequence context

ABCE1 (Rli1 in yeast) is a conserved ABC-type protein that plays a crucial role in translation termination and ribosome recycling ^3^. Recent studies have reported that the loss of ABCE1 caused ribosome stalling at stop codons and increased ribosome occupancy in 3’UTRs ^12^. To investigate whether ABCE1 has any sequence preference towards terminating ribosomes, we knocked down ABCE1 in HEK293 cells using shRNA (Figure S4A). As expected, silencing ABCE1 reduced cell proliferation and global protein synthesis (Figure S4B). We then conducted ribosome profiling in ABCE1 KD and control cells using Ezra-seq. When the ribosome occupancy in the CDS was normalized, loss of ABCE1 led to a modest increase of stop codon peaks (Figure S4C). Notably, the elevated ribosome density occurred at all stop codons, an indication of global effects. A closer look revealed that silencing ABCE1 increased the ribosome density at the −15 nt position but not the forward shifted one at the −12 nt position (Figure S4D), suggesting a delayed pre-termination in the absence of ABCE1. A previous study reported that ABCE1 knockdown in HeLa cells promoted 3’UTR translation in all reading frames ^12^. We could not confirm this feature partly due to the limited sequencing depth of our Ribo-seq. Another possibility is the incomplete ABCE1 knockdown in HEK293 cells (Figure S4A). We then employed the HiBiT-based 3’UTR reporter assay, which showed increased 3’UTR translation regardless of the sequence context upstream of stop codons (Figure S4E). Therefore, the ribosome splitting factor ABCE1 plays a generic role in translation termination with little sequence specificity.

### The 3’ end of 18S rRNA influences termination pausing

**Fig. 5.**
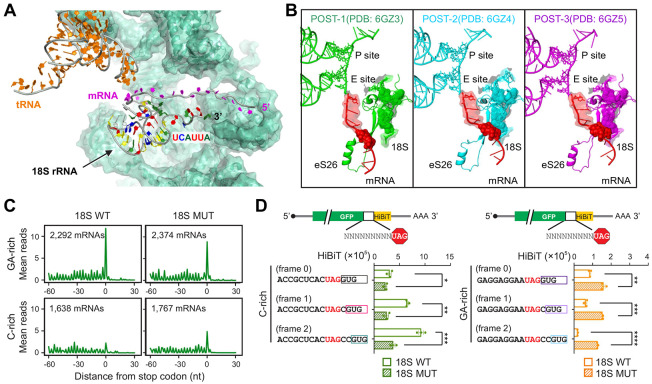
The 3’ end of 18S rRNA influences the dynamic of terminating ribosome. **(A)** A cryoEM structures of mammalian initiating ribosomes (PDB: 6ZMW) showing the proximity of the 3’ terminus of 18S rRNA and mRNA. **(B)** Superposition of different conformations sampled by normal mode analysis (NMA) of the region mentioned in (A). The analysis was carried out with the mRNA segment from the −10 nt to the +5 nt related to the A-site. The superposition is outlined only for the Rps26 protein (green surface), the mRNA segment (blue surface) and the last 10 nucleotides at the 3’ rRNA extremity (dark red surface), with the remaining proteins and rRNA segments at the site described only by the average structure (silver transparent surface and cartoon). The tRNA anticodon loop at the P ribosomal site is shown in dark cyan. **(C)** HEK293 cells were transfected with plasmids encoding 18S rRNA WT or mutant followed by Ribo-seq. Aggregation plots show the mean reads around stop codons of mRNAs with the GA sequence motif or C-rich sequence element. **(D)** HEK293 cells were transfected with plasmids encoding 18S rRNA WT or mutant followed by transfection with mRNA reporters shown in (4D). Bar graphs show the HiBiT signals in transfected cells with HiBiT at different reading frames. Error bars, mean ± s.e.m. *n* = 3 biological replicates. *P ≤ 0.05; **P ≤ 0.01; ***P ≤ 0.001; ****P ≤ 0.0001 by unpaired two-tailed *t*-test.

For a terminating ribosome, the mRNA sequence preceding the stop codon is positioned upstream of the E-site. Given the narrow mRNA channel, external factors like ABCE1 are unlikely to regulate ribosome behavior in a sequence-specific manner. Previous studies using crosslinking reported that mRNA near the exit site interacted with the 3’ terminus of 18S rRNA ^31^. The proximity between mRNA and 18S rRNA was also evident in recent cryoEM structures of mammalian initiating ribosomes ^32^, albeit the base pairing is not immediately clear (Figure 5A). The juxtaposition of mRNA and the 3’ end of 18S rRNA also holds true for elongating ribosomes undergoing translocation ^33^. Our modeling based on cryoEM structures suggest that, from the early to the late translocation intermediates (i.e., from the POST-1 to the POST-3), there is gradual approximation between mRNA (the region of − 9 to − 3) and the 3’ end of rRNA (Figure 5B). It is thus likely that mRNA undergoes post-decoding scanning by 18S rRNA. Since the 3’ end of 18S rRNA contains a highly conserved U-rich sequence (GAUCAUUA), the GA-rich sequence element of mRNA could follow U:A and U:G base pairing near the exit site (Figure 5A and S5A). By contrast, the C-rich sequence motif on mRNA would escape the 18S rRNA checkpoint, resulting in faster mRNA passthrough.

To examine whether the putative mRNA: rRNA base pairing contributes to termination pausing, we attempted to alter the 3’ end sequence of 18S rRNA. The presence of hundreds of rDNA copies in cells prevents us from using genome editing tools like CRISPR/Cas9. Instead, we used a previously described 18S rRNA expression system that has been shown to be able to incorporate the exogenously expressed 18S rRNA into ~15% of 40S subunits in transfected cells^34,35^. We constructed an 18S rRNA mutant by replacing the last two U residues with G (GAUCAGGA), which would switch the base pairing from the GA-sequence to the C-rich sequence. Compared to the wild type 18S, overexpression of the 18S mutant in HEK293 cells had similar polysome formation (Figure S5B). We then conducted ribosome profiling using Ezra-seq and observed comparable ribosome occupancy in the coding region (Figure S5C). When mRNAs are stratified based on the sequence motif upstream of stop codons, we found that overexpression of the 18S mutant reduced the differential termination pausing between GA-rich and C-rich sequences (Figure 5C). Specifically, mRNAs containing the GA-sequence motif showed reduced stop codon peaks in cells expressing the 18S rRNA mutant, whereas the C-rich sequence started to show elevated ribosome density at stop codons. The switch of termination pausing from the GA-sequence to the C-rich sequence in response to 18S rRNA mutation strongly suggests that the sequence specificity of termination pausing stems from the interaction between mRNA and the 3’ end of 18S rRNA.

To substantiate this finding further, we employed the HiBiT-based reporter assays to examine 3’UTR translation in cells expressing 18S WT or mutant. Overexpression of the18S mutant attenuated the 3’UTR random translation in reporters bearing the C-rich sequence (Figure 5D). For reporters containing the GA-sequence, we observed higher HiBiT signals, a sign of increased 3’UTR translation. Together with the swapped termination pausing by the 18S rRNA mutant, these results suggest a crucial role for the 3’ end of 18S rRNA in termination fidelity. Notably, the 3’ end sequence of 18S rRNA is highly conserved (Figure S5D). In the human genome, annotated stop codons are predominantly preceded by the GA-rich sequence (Figure S5E). By contrast, out-of-frame stop codons show a higher percentage of C-rich sequences. This finding suggests an evolutionary benefit for termination pausing at annotated stop codons, presumably by minimizing stop codon readthrough.

### Tissue-specific termination pausing

**Fig. 6.**
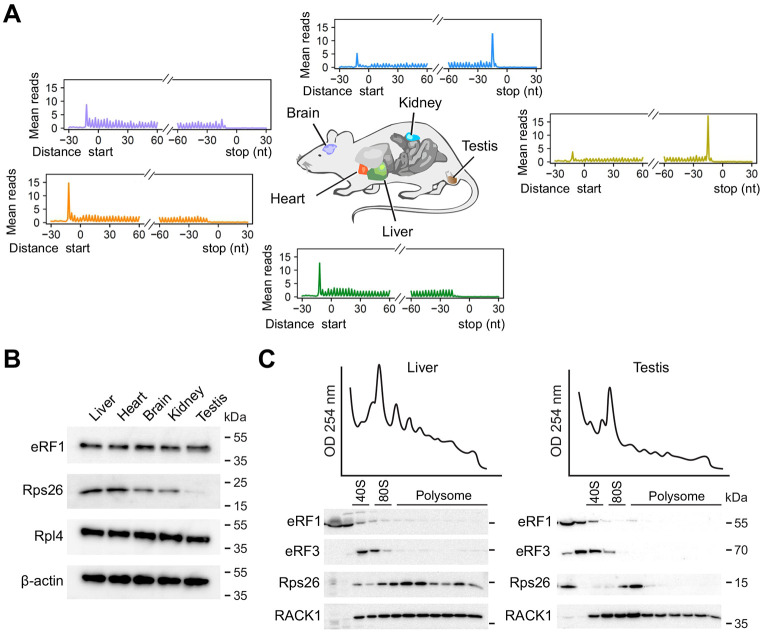
Differential termination pausing in mouse tissues. **(A)** Different mouse tissues were collected followed by Ribo-seq. Metagene analysis shows the distribution of mean ribosome reads across the transcriptome aligned to start and stop codons. **(B)** Representative Western blots of different mouse tissues using antibodies indicated. The experiment was independently repeated three times with similar results. **(C)** Mouse liver and testis were subjected to polysome profiling using sucrose gradient. Representative Western blots of ribosome fractions were conducted using antibodies indicated. The experiment was independently repeated three times with similar results.

At this point, we demonstrated sequence-specific termination pausing using cell lines in culture. To explore the physiological significance of termination pausing, we conducted Ribo-seq using a wide range of mouse tissues such as liver, brain, kidney, heart, and testis (Figure 6A). Different tissues were manifested by varied polysome patterns as well as the ribosome occupancy in the coding region (Figure 6A and S6A). Intriguingly, ribosome density at start and stop codons showed the largest variation in a reciprocal manner. While testis showed a small peak at start codons, there was a prominent peak at stop codons (Figure 6A). By contrast, termination peaks were barely observable in liver, heart, and brain, although these tissues showed evident initiation peaks. This was not due to differential gene expression as the same pattern maintains when the genes commonly or uniquely expressed in liver and testis were considered (Figure S6B). Further supporting the sequence specificity of termination pausing, testis mRNAs with prominent stop codon peaks are enriched with GA-sequences upstream of the stop codon (Figure S6C). The same group of mRNAs, however, barely exhibit termination pausing in liver. The diminished stop codon peak in mouse liver was reported in independent studies ^36^, excluding the possibility of technical bias of Ezra-seq.

The unexpected tissue-specific termination pausing suggests a mechanism beyond the sequence context in controlling the dynamics of terminating ribosomes. Notably, the eukaryote-specific ribosomal protein S26 (Rps26) also interacts with the mRNA region upstream of the E-site ^31,37^. Recent cryo-EM structures confirmed that both Rps26 (AA62 – 70) and 18S rRNA (nt1857 – 1863) constitute the mRNA path near the exit site (Figure 5B). Since Rps26 is positioned between mRNA and the 3’ end of 18S rRNA, we reasoned that absence of Rps26 could facilitate 18S rRNA: mRNA interaction. Interestingly, a recent study reported that Rps26 could dissociate from the fully assembled 80S ribosome in response to stress conditions, giving rise to ribosome heterogeneity ^38^. To examine the Rps26 variation in different tissues, we compared ribosomal proteins in tissue homogenates. Relative to β-actin, ribosomal proteins like Rpl4 showed comparable levels in all the tissues. However, testis showed the least amount of Rps26 among the tissues examined (Figure 6B). The differential protein levels of Rps26 in liver and testis also hold true in the Human Protein Atlas (proteinatlas.org) ^39^. We further assessed the Rps26 distribution in polysome fractions in liver and testis. While liver exhibited a similar distribution of Rps26 and RACK1 in polysome fractions, testis showed an evident depletion of Rps26 in polysome (Figure 6C). Notably, a substantial amount of Rps26 is present in the ribosome-free fraction of testis. Whether there is an active dissociation of Rps26 from translating ribosomes in testis awaits further investigation. These results nevertheless suggest the crucial role of Rps26 stoichiometry in termination pausing.

### Rps26 modulates termination pausing

**Fig. 7.**
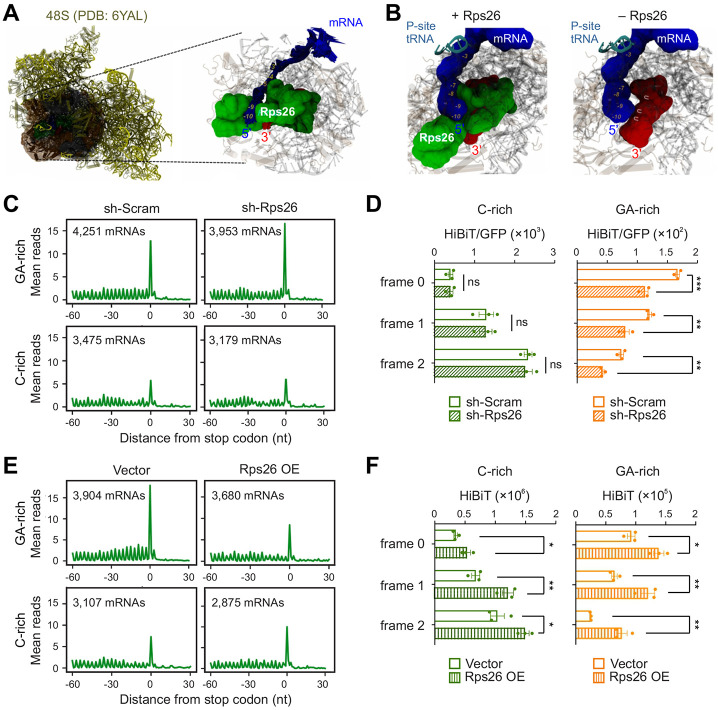
Rps26 modulates termination pausing. **(A)** A cryo-EM structure of the human 48S pre-initiation complex (PDB: 6YAL) depicted in cartoon and colored in yellow. The region at the 5’ exit of the mRNA tunnel centered in Rps26 and extended around 30 Å of this protein is highlighted in transparent surface and in dark colors. **(B)** Conformational sampling by normal mode analysis (NMA) of the region mentioned in (A). The analysis was carried out with the mRNA segment from the −10 nt related to the P-site to the +5 nt related to the A-site. The superposition is outlined only for the Rps26 protein (green surface), the mRNA segment (blue cartoon) and the last 10 nucleotides at the 3’ rRNA extremity (dark red cartoon), with the remaining proteins and rRNA segments at the site described only by the average structure (silver transparent surface and cartoon). The tRNA anticodon loop at the P ribosomal site is omitted on the image. **(C)** HEK293 cells with or without Rps26 knockdown were subjected to Ribo-seq. Aggregation plots show the mean reads around stop codons of mRNAs with the GA sequence motif or C-rich sequence element. **(D)** HEK293 cells with or without Rps26 knockdown were transfected with mRNA reporters shown in 4D. Bar graph shows the HiBiT signals at different reading frames upon normalization to upstream GFP levels Error bars, mean ± s.e.m. *n* = 3 biological replicates. ns, nonsignificant; **P ≤ 0.01; ***P ≤ 0.001 by unpaired two-tailed *t*-test. **(E)** HEK293 cells with or without Rps26 overexpression were subjected to Ribo-seq. Aggregation plots show the mean reads around stop codons of mRNAs with the GA sequence motif or C-rich sequence element. **(F)** HEK293 cells with or without Rps26 overexpression were transfected with mRNA reporters shown in 4(D). Bar graph shows the HiBiT signals at different reading frames. Error bars, mean ± s.e.m. *n* = 3 biological replicates. *P ≤ 0.05; **P ≤ 0.01 by unpaired two-tailed *t*-test.

Based on the available structures of ribosomes with bound mRNA, Rps26 is sandwiched between mRNA and the 3’ end of 18S rRNA (Figure 7A). We hypothesize that absence of Rps26 would facilitate broader mRNA:rRNA interaction, thereby promoting termination pausing. Indeed, normal mode analysis (NMA) by anisotropic network models suggests that, in the absence of Rps26, both the −3 to −9 extension of the mRNA and the 3’ end of 18S rRNA can twist and approximate to each other with improved mutual parity (Figure 7B). Such parity would be enhanced when the mRNA sequence is complementary to the 3’ end of 18S rRNA. Given the U-rich sequence at the 3’ end of 18S rRNA, such an interaction is more stable when the mRNA contains GA-rich sequences.

To investigate whether Rps26 haploinsufficiency affects ribosome dynamics at stop codons, we knocked down Rps26 from HEK293 cells using shRNA (Figure S7A). Rps26 silencing resulted in an increased 60S peak and reduced polysome formation (Figure S7B), which is consistent with its role in 40S subunit maturation ^40^. We then conducted ribosome profiling in parallel with RNA-seq. Intriguingly, Rps26 knockdown reduced the ribosome density at start codons but elevated the ribosome occupancy at stop codons (Figure S7C). The reciprocal change echoes the tissue-specific differences in initiation and termination (Figure 6A). The increased termination pausing, once again, primarily occurs at stop codons preceded with GA-rich sequences (Figure 7C). To examine the termination fidelity, we conducted HiBiT-based 3’UTR reporter assay. Since Rps26 silencing caused general reduction of protein synthesis (Figure S7B), we normalized HiBiT signals with upstream GFP levels. Only reporters with the GA-sequence, but not the C-rich sequence, showed reduced HiBiT translation in cells lacking Rps26 (Figure 7D). Therefore, Rps26 contributes to sequence-specific termination fidelity by controlling mRNA:rRNA interaction.

To substantiate this finding, we overexpressed Rps26 in HEK293 cells (Figure S7D), which did not affect polysome formation (Figure S7E). Ribosome profiling revealed that Rps26 overexpression lowered the averaged ribosome density from start to stop codons (Figure S7F). Remarkably, when mRNAs are stratified based on the sequence motif upstream of stop codons, we found that overexpression of Rps26 reduced the ribosome density (>50%) at stop codons preceded with the GA-sequence (Figure 7E). Notably, ribosome reads upstream of these stop codons were also depleted, suggesting a broader effect of Rps26 overexpression in elongating ribosomes. Further supporting the sequence-specific feature, mRNAs bearing the C-rich sequence before the stop codon showed higher termination peaks. To affirm the mechanistic connection between stop codon pausing and termination fidelity, we conducted HiBiT reporter assays that showed increased 3’UTR translation in cells with Rps26 overexpression (Figure 7F). Collectively, the functional consequence of mRNA:rRNA interaction is influenced by Rps26 stoichiometry, whose variation explains the tissue-specific translational control at stop codons.

## DISCUSSION

Translation termination is a relatively slower process compared to elongation; hence, stop codons themselves often act as ribosome pausing sites. Without pausing at stop codons, terminating ribosomes are likely to undergo incomplete dissociation, resulting in continuous translation in 3’UTR. Prolonged stalling, however, would lead to delayed ribosome recycling and collision of elongating ribosomes. Therefore, the ribosome dwell time at stop codons needs to be calibrated to ensure termination efficiency and accuracy. Since the development of ribosome profiling, the elevated ribosome density at both start and stop codons has been well-documented from metagene analysis. However, very little is known about the variations of termination pausing at individual stop codons, let alone the underlying mechanism. With the help of high-resolution ribosome profiling ^22^ and specialized eRF1-seq, we quantified stop codon pausing indexes and uncovered the potential role of sequence contexts in the dynamics of terminating ribosomes. Unexpectedly, it is the sequence upstream of the stop codon that controls termination pausing. The pausing-associated GA-sequence element is further confirmed by massively paralleled reporter assay. By contrast, the C-rich sequence motif eliminates termination pausing when present before the stop codon. Importantly, lack of termination pausing leads to stop codon-associated random translation, giving rise to mixed C-terminal extension. This phenomenon differs from stop codon readthrough and implies more complex regulation of terminating ribosomes.

One potential pitfall in interpreting the sequence-specific termination quality control is the different amino acids encoded by the sequences before the stop codon. This is particularly true for C-rich sequences that tend to encode proline. However, several lines of evidence suggest that it is the nucleotide sequence that controls ribosome dynamics at stop codons. First, the positional effect of sequence elements does not follow codon triplets. Second, while the proline codon is slow to decode, the C-rich sequence reduces termination pausing. Third, the C-rich sequence-promoted readthrough occurs even when proline codons are avoided. Fourth, fusion proteins showed minimal effect of those codons on translation. Fifth, the unbiased MPRA revealed the enrich sequences in all reading frames. We conclude that it is the mRNA sequences rather than the encoded amino acids that contribute to ribosome dynamics at stop codons.

Since the sequence upstream of the stop codon is positioned near the exit site of the mRNA channel, it is possible that the communication between ribosome and mRNA continues beyond the decoding center. The putative mRNA:rRNA interaction is quite common during translation initiation, which is exemplified by the Shine-Dalgarno (SD) sequence that pairs with the 3’ end of 16S rRNA in prokaryotic cells ^41^. In eukaryotic cells, a similar base pairing has been documented to facilitate cap-independent translation and cap-dependent translation of histone H4 mRNA ^42-44^. We previously reported that the highly conserved 3’ end of 18S rRNA contributes to elongation pausing via base pairing with certain codons ^35^. It is likely that the same feature extends to terminating ribosomes, resulting in termination pausing in a sequence-specific manner.

Although ribosomal structures have been deciphered at atomic levels, only a few available structures contain mRNA. Due to the flexible nature of the linear mRNA, the entire mRNA path within the channel was partially resolved. Although crosslinking experiments indicate proximity between mRNA and rRNA near the exit site ^31^, the physiological significance remains unclear. Using 18S rRNA mutants, we found that the U-rich sequence at the 3’ end of 18S rRNA is crucial in holding mRNAs bearing the GA-sequence. The putative G:U base pair is particularly interesting as it exhibits unique chemical, structural, and dynamic properties compared to the A:U base pair ^45^. When the U-sequence was mutated to G, we observed a switch of termination pausing from GA-sequence to C-rich sequence on mRNAs. Therefore, the mRNA:rRNA base pairing near the exit channel potentially delays the mRNA movement after decoding. For terminating ribosomes, the prolonged dwell time at stop codons offers an extended window for eRF1 loading, peptide cleavage, and ribosome recycling.

The post-decoding mRNA:rRNA interaction, albeit attractive, does not explain tissue-specific variations of termination pausing. The strong termination pausing in testis is particularly interesting. Compared to other types of tissues, the testis transcriptome has the highest diversity and complexity due to the promiscuous transcriptional activity ^46^. Testis is also known as the birthplace of new genes during evolution by offering a more permissive environment with less stringent nonsense-mediated decay (NMD) pathways ^47^. Besides the widespread transcription and attenuated RNA decay, the mRNA translation process in testis is also unique. For instance, translation efficiency is less influenced by the codon optimality ^48^. Additionally, 3’UTR translation in spermatocytes is coupled with piRNA biogenesis ^49^. It is thus conceivable that the wide range of ribosome density at stop codons in testis facilitates functional division of ribosome occupancy beyond the coding region. Whether the testis exhibits distinct ribosome dynamics at stop codons during spermatogenesis awaits further investigation.

To search for cell type-specific regulators of termination pausing, we focus on the eukaryotic-specific Rps26 that was likely evolved for distinct cellular needs. Like the 3’ end of 18S rRNA, Rps26 readily crosslinked to mRNA regions upstream of the P-site codon ^31,37^. In fact, a central fragment of Rps26 is located beneath an mRNA region 5’ of the E-site codon, thereby blocking the direct interaction of this mRNA segment with the 3’ end of 18S rRNA (Figure 7A). It is likely that Rps26 removal promotes 18S rRNA and mRNA base pairing. Indeed, silencing Rps26 further elevated ribosome density at stop codons with upstream GA-sequences, whereas Rps26 overexpression dampened termination pausing. A previous study reported that Rps26 directs mRNA-specific translation by recognition of Kozak sequence elements ^50^. Notably, the consensus Kozak sequence context in vertebrates is GCCRCCAUGG, where R is a purine (A/G). Among the C-rich sequence upstream of AUG, the importance of purine at −3 position is widely appreciated. Intriguingly, we observed a reciprocal pattern between initiation and termination peaks in cells lacking Rps26, suggesting that the functional interactions between mRNA, rRNA, and Rps26 occur at all three stages of translation.

Perhaps the most surprising finding in our study is the tissue-specific termination pausing controlled by the Rps26 stoichiometry. In yeast cells, Rps26 could dissociate from fully assembled 80S ribosomes in response to stress conditions ^38^. It is possible that, in mammalian cells, Rps26 is either sub-stoichiometric or loosely integrated into the 80S ribosomes. Notably, the haploinsufficiency of Rps26 is linked to the pathogenesis of Diamond-Blackfan anemia (DBA) ^51^. The prolonged termination pausing likely blocks ribosome recycling, explaining the hematologic phenotypes of DBA. Compared to other cell types, erythroid cells have a high demand of the ribosome rescue pathway and exhibit unusually high 3’UTR translation ^52^. A better understanding of the stop codon-associated translational quality control could lead to the development of therapeutic targets towards DBA or other nonsense mutations of inherited human diseases.

## STAR★METHODS

### RESOURCE AVAILABILITY

#### Lead contact

All material request should be directed to Shu-Bing Qian (sq38@cornell.edu).

#### Materials availability

Reagents and materials produced in this study are available from the Lead Contact pending a completed Materials Transfer Agreement.

#### Data and code availability

- All Sequencing data are available in the Gene Expression Omnibus database. All raw images are available in Mendeley Data. All data are publicly available as of the date of publication. Accession numbers and DOI are listed in the key resources table.
- All custom code has been deposited to GitHub and Zenodo. DOI are listed in the key resources table.
- Any additional information required to reanalyze the data reported in this paper is available from the lead contact upon request.

### EXPERIMENTAL MODEL AND SUBJECT DETAILS

#### Cell lines

HEK293-K^b^ cells and Lenti-X 293T cells are maintained in Dulbecco’s Modification of Eagle’s Medium (Corning, 10-013-CV) with 10% fetal bovine serum (Sigma, 12306C). All cells were grown at 37°C with 5% CO_2_.

#### Mouse strains

C57BL/6 mice were obtained from the Jackson laboratory. All animals (1-6 mice per cage) were housed in a 12 h light/dark cycle in the Weill Hall animal facility at Cornell University with the supervision of the Center for Animal Resources and Education (CARE) breeding program. All animals used in this study were handled in accordance with federal and institutional guidelines, under a protocol approved by the Cornell University Institutional Animal Care and Use Committee, protocol 2017-0035. Mice were housed under specific pathogen-free conditions in an Association for the Assessment and Accreditation of Laboratory Animal Care International-accredited facility and cared for in compliance with the Guide for the Care and Use of Laboratory Animals.

### METHOD DETAILS

#### Antibodies

The following antibodies were used at their indicated experimental concentrations: anti-GFP (Proteintech, 50430-2-AP, 1:1000), anti-eRF1 (Santa Cruz Biotechnology, sc-365686, 1:200), anti-eRF3 (Santa Cruz Biotechnology, sc-515615, 1:200), anti-Rps26 (Proteintech, 14909-1-AP, 1:500), anti-Rpl4 (Proteintech, 11302-1-AP, 1:1000), anti-ABCE1 (Abcam, ab185548, 1:1000), anti-myc (Cell Signaling, 2272S, 1:1000), anti-RACK1 (BD Transduction Laboratories, 610177, 1:1000) and anti-β-Actin ((Sigma-Aldrich, A5441, 1:5,000). anti-mouse IgG horseradish peroxidase (HRP)-conjugated secondary antibodi (Sigma-Aldrich, A0168, 1:10,000) or antirabbit IgG secondary antibody conjugated to peroxidase (Sigma-Aldrich, A9169, 1:10,000).

#### Plasmid construction

The template of EGFP-HiBiT reporters were PCR-amplified from the pcDNA3-EGFP vectors using reverse primer containing the desired sequence. In some cases, the template of reporters based on HiBiT-EGFP were generated using a two-step PCR amplification approach. First, the full length of HiBiT and EGFP was amplified from pcDNA3-EGFP to generate HiBiT-EGFP. The resulting PCR product was used as a template to produce the full-length reporter using a second forward primer containing the respective sequence. After column purification (QIAGEN), the DNA template (1~2 μg) was utilized to generate mRNAs suitable for transfection. For exogenous Rps26, the full-length coding sequence of human Rps26 was cloned into pcDNA3.1(myc-His B) using BamH I and Hind III restriction sites. To create the 18S rRNA mutant, site-directed mutagenesis was performed using Q5 Site-Directed Mutagenesis Kit (New England Biolabs) according to the manufacturer manual. Mutation was confirmed by Sanger DNA sequencing. DNA sequences of all primers used in this study are listed in the Key Resources Table.

#### In vitro transcription

To prepare mRNA reporters, 1~2 μg PCR products described above were utilized as templates to generate mRNAs suitable for transfection. In vitro transcription was performed for 1 h at 37 °C using mMESSAGE mMACHINE T7 Transcription Kit (Invitrogen) followed by poly(A) tailing (Ambion) at 37 °C for 30 min. The resulting RNAs were purified using RNA Clean & Concentrator (Zymo Research) following the manufacturer’s instruction and stored at −80 °C.

#### Transfection

For mRNA reporter transfection, cells were transfected with in vitro transcribed mRNA (1 μg) in Opti-MEM (125 μl) using Lipofectamine MessengerMAX (1 μl) in Opti-MEM (125 μl), unless stated otherwise. The cells were incubated with the mRNA/Lipofectamine MessengerMAX mixture for 4 h followed by immunoblotting, HiBiT assay or polysome profiling. For Rps26 or 18S rRNA overexpression, 2 μg plasmids were mixed with 4 μl Lipofectamine 2000 (Invitrogen) followed by incubation with cells for at least 24 h, unless stated otherwise.

#### HiBiT assay

Cells grown in a 35 mm dish were transfected with mRNA reporters (1 μg) described above. Transfected cells were washed with PBS and then lysed using a Nano-Glo HiBiT Lytic Detection System (Promega) according to manufacturer’s instructions. HiBiT signals were measured using Luminometer (Atto).

#### Immunoblotting

Cells grown in a 6-well plate were transfected with transcribed mRNA reporters (1 μg) described above. Transfected cells were washed twice with ice-cold PBS and lysed on ice by adding SDS-PAGE sample buffer (50 mM Tris pH 6.8, 100 mM DTT, 2% SDS, 0.1% bromophenol blue, 10% glycerol), followed by heating for 10 min at 95 °C. Protein samples were separated on SDS-PAGE gels followed by transferring to PVDF membranes (Thermo Fisher Scientific). Membranes were blocked in 5% non-fat milk (Bio-Rad) in TBS containing 0.1 % Tween-20 (TBST) for 1 h, followed by incubation overnight with primary antibodies at 4 °C. After 3 × 10 min washes in TBST, membranes were incubated with anti-mouse IgG horseradish peroxidase (HRP)-conjugated secondary antibodies at room temperature for 1 h. Membranes were then washed 3 × 10 min in TBST at room temperature and visualized using chemiluminescence by exposing to ECL film (GE Healthcare).

To prepare tissue lysates, mouse tissues were dissected and snap-frozen in liquid nitrogen. Frozen tissues were thawed and homogenized on ice with homogenizer (U.S. Solid) in ice-cold RIPA buffer (150 mM NaCl, 50 mM Tris-HCl pH 8.0, 1% Triton X-100, 0.5% sodium deoxycholate, 0.1% SDS) with 1 × protease inhibitors (Roche). After centrifugation at 16,000 g for 20 min at 4 °C, supernatants were collected for immunoblotting as described above. Proteins in mouse tissue fractions collected from sucrose density gradients were precipitated by trichloroacetic acid (TCA) and resuspended in 2 × SDS-containing sample buffer. To detect HiBiT-tagged proteins by Nano-Glo HiBiT blotting system (Promega), cell lysates were resolved by SDS-PAGE and transferred to PVDF membranes as described above. The membrane was incubated with 1 × TBST for 1 h at room temperature, followed by incubation in the LgBiT/buffer solution (50 μl of LgBiT protein in 10 ml of Nano-Glo blotting buffer) at room temperature for 1 h. 20 μl of substrate was added to the incubation solution for additional 5 min. The membrane was exposed to ECL film in the same manner as the immunoblot analysis.

#### shRNA knockdown

shRNAs targeting Rps26 and ABCE1 were designed from BROAD RNAi consortium database and subcloned into DECIPHER pRSI9-U6-(sh)-UbiC-TagRFP-2A-Puro (Cellecta). A scrambled shRNA was used as control. Lentiviral particles were produced using Lenti-X 293T cells (Clontech). The supernatants containing viral particles were harvested at 48 h after transfection and filtered through a 0.45 μM Millex-HP filter unit (Millipore). HEK293 cells were transduced with shRNA lentivirus for 48 h followed by selection with 2 μg/ml puromycin. Knockdown efficiency was detected by immunoblotting using indicated antibodies. The oligonucleotide sequences are listed in the Key Resources Table.

#### Polysome profiling

A total of 4 plates (10-cm) HEK293 cells grown to 80% confluency were washed by cold PBS and lysed in the polysome lysis buffer (10 mM HEPES, pH 7.4, 100 mM KCl, 5 mM MgCl_2_, 100 μg/mL cycloheximide with 1% Triton X-100). The nuclei were pelleted by spinning at 14,000 rpm for 10 min at 4 °C. For mouse tissues, 100 mg of frozen samples were homogenized on ice using a Dounce homogenizer in 1 mL polysome lysis buffer. Homogeneous lysates were cleared by centrifugation at 14,000 rpm for 10 min at 4 °C. 500 μL of lysates were loaded onto a 15-45% (wt/vol) sucrose density gradients freshly prepared in a SW41 ultracentrifuge tube (Backman) using a Gradient Master (BioComp Instruments). Samples were centrifuged at 180,000 g for 2 h 30 min at 4 °C in a Beckman SW41 rotor. Polysome profiles were recorded at A254 using the Brandel Gradient Fractionation System and an ISCO UA-6 UV/Vis detector.

#### Ribosome profiling

The Ezra-seq has been described previously ^22^. In brief, an aliquot of ribosome fractions representing monosome or polysome were collected followed by digestion with *E. coli* RNase I (Ambion, 750 U per 100 A260 units) by incubation at 4 °C for 1 h. RNA was extracted using Trizol LS reagent (Invitrogen) followed by ethanol precipitation. The ribosome-protected mRNA fragments (RPFs) were separated on a 15% polyacrylamide TBE-urea gel (Invitrogen) and visualized using SYBR Gold (Invitrogen). Selected regions in the gel corresponding to 25-35 nt were excised and dissolved by soaking in 400 μl RNA elution buffer (300 mM NaOAc pH 5.2, 1 mM EDTA, 0.1 U/μl SUPERase·n) for 10 min at 70 °C. The gel debris was removed using a Spin-X column (Corning), followed by ethanol precipitation. 14 μl RNAs (10~200 ng) were mixed with 1 μl T4 PNK (NEB), 20 U SUPERase·In in 1 × T4 PNK buffer and incubated at 37 °C for 30 min followed by 65 °C for 20 min. After ethanol precipitation, 10 μl dissolved RNA were mixed with 1 μl homemade Ezra enzyme, 1 μl Poly(A) Polymerase (NEB) and 20 U SUPERase·In in 7 μl Ezra buffer. After incubation at 37 °C for 30 min followed by 65 °C for 20 min, 1 μl of 1 μM 5’ end adaptor, 1 μl of 1 μM biotinylated reverse transcription primer, 20 U SUPERase·In were added and incubated at 70 °C for 3 min followed by slowly cooling down (3 °C/min) to 25 °C. The hybridized RNA sample was mixed with 10 μl of pre-washed streptavidin beads and incubated at room temperature for 10 min. Beads were washed and re-suspended in 10 μl nuclease-free water. Ligation was performed for 60 min at 25 °C by mixing beads with a 10 μl reaction mixture (1 × T4 Rnl2 reaction buffer, 20 U SUPERase·In, 15% PEG8000 and 200 U T4 RNA ligase 2 truncated KQ (NEB)). After washing once with 2 × SSC, beads were re-suspended in 12 μl nuclease-free water and mixed with 8 μl cDNA synthesis mixture (5 × first strand buffer, 0.1 M DTT, 10 mM dNTP, RNaseOUT and SuperScript III) followed by incubation at 50 °C for 30 min. After washing once with 2 × SSC, beads were resuspended in 10 μl nuclease-free water and incubated at 95 °C for 2 min, then immediately placed on the ice for 1 min. After placing on magnet stand for 1 min, the supernatant cDNA was amplified by PCR using barcoded sequencing primers. PCR was performed by mixing 1 × HF buffer, 0.5 mM dNTP, 0.25 μM PCR primers and 0.025 U Phusion polymerase. PCR was carried out under the following conditions: 98 °C, 30 s; (98 °C, 5 s; 68 °C, 15 s; 72 °C, 10 s) for 12 cycles; 72 °C, 3 min. PCR products were separated on a 8% polyacrylamide TBE gel (Invitrogen). DNA products with the expected size 180 bp were excised and recovered from DNA elution buffer (300 mM NaCl, 1 mM EDTA). After quantification by Agilent BioAnalyzer DNA 1000 assay, equal amounts of barcoded samples were pooled and sequenced using NextSeq 500 (Illumina). The oligonucleotide sequences are listed in the Key Resources Table.

#### eRF1-seq

A total of 4 plates (10-cm) HEK293 cells with 80% confluence were washed three times with ice-cold DPBS. Cells were fixed in ice-cold formaldehyde solution (0.5% in DPBS) for 10 min at 4 °C on a rocker. After washing with ice-cold DPBS three times, cells were quenched in ice-cold buffer (50 mM Glycine, 50 mM Tris-HCl pH 7.5 in nuclease-free water) for 10 min at 4 °C on a rocker. Cells were then washed with polysome buffer and lysed in the 400 μl of polysome lysis buffer with 1% Triton-X-100 on ice. Cell debris was removed by centrifugation at 15,000 rpm for 10 min at 4 °C. The supernatant was digested with RNase I (Ambion, 750 U per 100 A260 units) for 1 h at 4 °C. Digested supernatant was loaded onto sucrose gradients for polysome profiling as described above. The 80S fraction was collected (~200 μl total) and mixed with 10 μg eRF1 antibody and 0.5 U/μl SUPERaseÎn (Invitrogen) followed by incubation under gentle rotation at 4 °C for 5 h. Protein A/G beads were added into the mixture and rotated at 4 °C overnight. Beads were washed three times and then resuspended in 600 μl of polysome buffer. RNA was extracted from resuspended beads in polysome buffer. Briefly, samples were brought to room temperature and then adjusted to 10 mM Tris-HCl pH 7.4, 10 mM glycine, 1% (w/v) SDS and 10 mM EDTA pH 8.0 followed by incubation at 65 °C for 5 min. One volume of acidic phenol/chloroform solution was added and vortexed at maximum speed for 2 min. Mixtures were then placed into thermomixer and shake at 1,400 rpm for 20 min at 65 °C to reverse the crosslinks. After centrifugation at 14,000 rpm for 5 min at 4 °C, the aqueous phase was precipitated with ethanol. Purified RNA was used for cDNA library construction and high-throughput sequencing as described above.

#### Massively paralleled reporter assay (MPRA)

From the PCR product of HiBiT-EGFP described above, a second PCR was conducted using the pooled oligonucleotide library (IDT) and a primer containing the T7 promoter. The DNA template (1~2 μg) was utilized to generate the mRNA library via in vitro transcription as described above. Cells with 80% confluence were transfected with 6 μg of mRNA library using Lipofectamine MessengerMAX. Cells were lysed 4 h after transfection followed by polysome profiling as described above. Fractions of 500 μl corresponding to monosome or polysome were collected for RNA extraction using TRIzol LS. RNA was purified using RNA Clean & Concentrator and eluted with 11 μl of nuclease-free water. The purified RNA was reverse transcribed using SuperScript III and gene-specific primers. In brief, RNA samples were mixed with 1 μl of 10 mM dNTP, 2 pmol reverse primer and incubated at 65 °C for 5 min, then immediately placed on ice for 1 min. The reverse transcription was carried out by incubating with the 7 μl reaction mixture (5 × first strand buffer, 0.1 M DTT, RNaseOUT and SuperScript III) at 50 °C for 60 min followed by heating at 70 °C for 15 minutes. The products were then amplified with Illumina-based sequencing primers with barcode. PCR were performed by mixing 1 × HF buffer, 0.5 mM dNTP, 0.25 μM PCR primers and 0.025 U Phusion polymerase. The PCR was initiated at 98 °C, 30 s; then (98 °C, 5 s; 68 °C, 15 s; 72 °C, 10 s) for 12 cycles; 72 °C, 3 min. The PCR products with the expected size 190 bp were excised from a 8% polyacrylamide TBE gel. The DNA products were recovered from DNA elution buffer, followed by quantification using Agilent BioAnalyzer DNA 1000 assay. Equal amounts of barcoded samples were pooled for sequencing using NextSeq 500 (Illumina). The oligonucleotide sequences are listed in the Key Resources Table.

#### Structural analysis

To gain insights about the relative orientations and interaction likelihood between the 5’ end of the mRNA (site −13 to −3 related to the A-site) and the 3’ end of the 18S rRNA along the ribosome translocation, a structural analysis was carried using the respective PDBs 6GZ3 (with a 3.60 Ǻ resolution), 6GZ4 (3.60 Å), 6GZ5 (3.50 Å) and 6yal (3.00 Å). The PDBs 6GZ3, 6GZ4 and 6GZ5 encompass three respective intermediate snapshots between the PRE and the POST translocation steps for the eukaryotic 80S ribosome (hereafter, referred simply as PRE and POST states). These three structures (hereafter referred as translocation intermediates POST 1 to 3, or simply TI-POST 1 – 3 for the respective PDBs 6GZ3 – 5) were solved, described and discussed previously ^33^. The mRNA environment sequentially described by these three PDBs provides a reasonable glimpse about the changes occurring after the recognition of at the A site codon and along the movement of the ribosome to the next codon. The PDB 6YAL, in turn, is the Homo sapiens 48S late-stage initiation complex ^53^. Due to the higher resolution of the PDB 6YAL, its mRNA structure is solved until a higher 5’ extension compared to the 6GZ3–5 PDBs (The mRNA 5’ in 6YAL starts from the −18 nt related to the site A, while in 6GZ3 it starts from the site −7 and from the site −6 in 6GZ4 and 6GZ5). In this way, we have made use of the 5’ fragment of the 6YAL mRNA structure to build a rigid body model of the same extension starting from the −13 nt at each one of the PDBs 6GZ3-5, using structural alignment of the respective backbone atoms in each oligonucleotide extremity in Pymol [Schrodinger, LLC. 2010. The PyMOL Molecular Graphics System, Version 2.5].

#### Anisotropic network modeling

The relative local fluctuations of the exit mRNA channel at the ribosome structure and the consequent proximity likelihood between the mRNA 5’ extension and the rRNA 3’ end was estimated in presence and absence of Rps26 by normal mode analysis (NMA) using anisotropic network modeling (ANM) ^54^. For this analysis, we used the atoms from the PDB 6YAL around a 30 Ǻ region centered on Rps26 (depicting the mRNA channel exit) both in the presence and absence of this protein. The PDB 6YAL was chosen due to its higher resolution as a whole, besides longer extension of the mRNA 5’ end compared to the other three PDBs structurally analyzed in this study. Furthermore, the region around 30 Ǻ from Rps26 (hereafter called Rps26 site) encompass a symmetric sphere composed basically of residues from the 40S subunit (including the 18S 3’ extension), mRNA (sites −13 to +5 nt related to the A-site) and the anticodon loop from the P-site tRNA, common to all the four structures here analyzed. Finally, this region in 6YAL presents a relatively small global root mean square deviation (RMSD), considering the protein and RNA backbone, related to both PDBs 6GZ3 and 6GZ5 (1.478 Å in the two cases). In this way, the PDB 6YAL was considered an accurate approximation of the general environment of the Rps26/mRNA 5’ end/rRNA 3’ end triad in ribosome, despite portraying a pre-initiation structure.

The ANM was carried using the ProDy tools ^55-57^. A Hessian matrix was built upon the backbone atoms of the Rps26 site for both proteins (Cα) and RNA (P, C4’ and C2) with and without Rps26 (hereafter referred as + Rps26 and − Rps26). The mRNA was considered from the −10 to the +5 nt, once the first three residues (−13 to −11) are more distant from the rRNA 3’ end and free of direct contacts with the neighborhood as a whole, which makes their movements dominate the NMA if they are considered on the ANM (not shown). The Hessian matrix was configured using the default parameters of distance cutoff and gamma function, with the respective values of 15 Å and 1.0 kcal/(mol.Å2). Initially, the first 50 normal modes of each system were estimated by obtaining their respective covariance matrixes by diagonalizing the Hessiam matrixes. The modes simultaneously containing the largest possible eigenvalues and higher mRNA fluctuations at the −10 to −3 extension (directly parallel to the rRNA 3’ end and separated from it by Rps26), as well as the fluctuations of the last 10 residues at the 18S 3’ end, were selected. In this way, the 15 first ANM normal modes estimated from the + Rps26 and − Rps26 systems were taken for conformational sampling analysis.

The +Rps26 and −Rps26 structures of the Rps26 site from above were taken to sampling of alternate conformations along the global fluctuations described by their respective 15 first ANM modes using ProDy ^55-57^. Basically, an extended ANM model containing all the protein and rRNA atoms for each structure was built from the original coarse grain model containing only the backbone atoms used to build the Hessian matrix. All the atoms at the extended model still obey the movements dictated by the selected 15 first normal modes, with each side chain atom moving in the same direction that the backbone atoms of the residue to which they belong. A set of 70 conformations symmetrically distributed along the fluctuation governed by the 15 first normal modes and with an average RMSD of 2.5 Å related to the input structure was sampled for each one of the +Rps26 and −Rps26 models. Finally, from each original 70 conformers set, a subset containing only the sterically feasible structures (i.e., without significant clashes or conformational distortions) was taken for analysis. Although higher refinements would be necessary to take these final conformers to rigorous molecular dynamics or free energy calculation studies, they provide enough insights about the Rps26 influence on the mRNA 5’ end/ 18S 3’ end fluctuations and interaction distance likelihood at the ribosome context.

### QUANTIFICATION AND STATISTICAL ANALYSIS

Data is presented as mean ± SEM, unless otherwise stated. At least three independent biological replicates have been performed for each experiment. The number of independent experiments is indicated. Statistical tests used and specific p-values are indicated in the figure legends.

#### Analysis of Ribo-seq and eRF1-seq

The adaptor of sequencing reads was clipped by Cutadapt, using parameters: -a AAAAAA --max-n=0.1 -m 15. The clean reads were then aligned to human transcriptome (GRCh38.81), which contains the protein coding transcripts with the longest CDS, using STAR with default parameters. To avoid ambiguity, reads mapped to multiple positions or with > 2 mismatches were disregarded for further analysis. Ribosome P-site was defined as the positions of 12th, 13th and 14th from 5’ end of the read (position 0). A-site was defined as the positions of 15th, 16th and 17th. To generate aggregation plot around the start and stop codons, for each mRNA, the aligned reads at individual sites were normalized by mean reads of the CDS. mRNAs with total reads in CDS < 16 or the CDS sites covered by footprints < 10% were excluded. The normalized values of the sites with the same distance relative to the start codon or stop codon were averaged across transcriptome.

#### Identification of termination peaks

To identify termination peaks, all reads of eRF1-seq were assigned to individual sites on mRNAs. The mRNAs with < 10 total reads from eRF1-seq were excluded. A 120-nt sliding window was used to scan along the mRNA, the sites with terminating reads tenfold higher than the average reads within the sliding window were defined as the termination peaks.

#### uORF prediction

For each mRNA, all possible uORFs starting with AUG were first extracted. A Wilcoxon test was applied to test whether the in-frame reads are significantly higher than the other two frames. The two *P* values were then combined to a single *P* value using a Stouffer’s method. uORFs with a false discovery rate (FDR) <0.05 were defined as the uORFs with robust translation.

#### RNA secondary structure analysis

A 30-nt sliding window was used to scan 3’ UTR. For each window, the minimum fold free energy (MFE) was calculated by ViennaRNA [PMID: 22115189] using default parameters.

#### Analysis of MPRA dataset

For each raw sequencing file, the adaptors at both ends were removed by cutadapt. The trimmed reads with length unequal to 9 nucleotides were excluded from analysis. The remaining trimmed reads were counted and then an RPM value (reads per million) was obtained by dividing the resultant read count by the total count.

## Supplementary Material

1

## Figures and Tables

**Table:** KEY RESOURCES TABLE

REAGENT or RESOURCE	SOURCE	IDENTIFIER
Antibodies		
Anti-GFP antibody	Proteintech	Cat# 50430-2-AP; RRID: AB_11042881
Anti-eRF1 antibody	Santa Cruz Biotechnology	Cat# sc-365686; RRID: AB_10843214
Anti-eRF3 antibody	Santa Cruz Biotechnology	Cat# sc-515615
Anti-Rps26 antibody	Proteintech	Cat# 14909-1-AP; RRID: AB_2180361
Anti-Rpl4 antibody	Proteintech	Cat# 11302-1-AP; RRID: AB_2181909
Anti-ABCE1 antibody	Abcam	Cat# ab185548; RRID: AB_2858278
Anti-myc antibody	Cell Signaling	Cat# 2272S
Anti-RACK1 antibody	BD Transduction Laboratories	Cat# 610177; RRID: AB_397576
Anti-β-Actin antibody	Millipore Sigma	Cat# A5441; RRID: AB_476744
Anti-Mouse IgG (Fc specific)-Peroxidase antibody	Millipore Sigma	Cat# A0168; RRID: AB_257867
Anti-Rabbit IgG (whole molecule)-Peroxidase antibody	Millipore Sigma	Cat# A9169; RRID: AB_258434
Bacterial and virus strains		
DECIPHER pRSI9-U6-(sh)-UbiC-TagRFP-2A-Puro	Cellecta	N/A
Subcloning Efficiency DH5a Competent Cells	Thermo Fisher Scientific	Cat# 18265-017
Chemicals, peptides, and recombinant proteins		
Dulbecco’s Modified Eagle Medium	Corning	Cat# 10-013-CV
Fetal bovine serum	Millipore Sigma	Cat# 12306C-500ML
Opti-MEM I Reduced Serum Medium	Thermo Fisher Scientific	Cat# 31985-070
Dulbecco’s Phosphate Buffered Saline	Thermo Fisher Scientific	Cat# 14190250
BamHI-HF	New England BioLabs	Cat# R3136S
HindIII-HF	New England BioLabs	Cat# R3104S
Lipofectamine MessengerMAX	Invitrogen	Cat# LMRNA015
Lipofectamine 2000	Invitrogen	Cat# 11668-500
DL-Dithiothreitol	Millipore Sigma	Cat# D0632-5G
Blotting-Grade Blocker	Bio-Rad	Cat# 1706404
Tween-20	Millipore Sigma	Cat# P7949-500ML
Triton-X100	Millipore Sigma	Cat# T9284-100mL
EDTA-free Protease Inhibitor Cocktail Tablets	Millipore Sigma	Cat# 11 836 170 001
Puromycin	Millipore Sigma	Cat# P7255
Cycloheximide	Millipore Sigma	Cat# C1988-1G
TRIzol LS Reagent	Invitrogen	Cat# 10296-028
Nuclease-Free Water	Invitrogen	Cat# AM9932
SUPERase·In RNase Inhibitor	Invitrogen	Cat# AM2696
RNase I	Invitrogen	Cat# AM2295
15% TBE-Urea Gels	Invitrogen	Cat# EC6885BOX
SYBR Gold nucleic acid gel stain	Invitrogen	Cat# S-11494
Sodium acetate buffer solution	Millipore Sigma	Cat# S7899-500ML
T4 Polynucleotide Kinase	New England BioLabs	Cat# M0201L
E. coli Poly(A) Polymerase	New England BioLabs	Cat# M0276L
T4 RNA Ligase 2, truncated	New England BioLabs	Cat# M0242L
Ezra enzyme	This paper	N/A
UltraPure^™^ SSC, 20X	Invitrogen	Cat# 15557044
Streptavidin Magnetic Beads	New England BioLabs	Cat# S1420S
RNaseOUT Recombinant Ribonuclease Inhibitor	Invitrogen	Cat# 10777-019
8% TBE Gel	Invitrogen	Cat# EC6215BOX
Pierce Protein A/G Agarose	Thermo Fisher Scientific	Cat# 20421
Critical commercial assays		
QIAquick Gel Extraction Kit	Qiagen	Cat# 28706
Q5 Site-Directed Mutagenesis Kit	New England BioLabs	Cat# E0554S
mMESSAGE mMACHINE T7 Transcription Kit	Invitrogen	Cat# AM1344
Poly(A) Tailing Kit	Invitrogen	Cat# AM1350
RNA Clean and Concentrator-25 Kit	Zymo	Cat# R1018
Nano-Glo HiBiT Lytic Detection System	Promega	Cat# N3040
Nano-Glo HiBiT Blotting System	Promega	Cat# N2410
SuperScript III Reverse Transcriptase	Thermo Fisher Scientific	Cat#18080-044
Deposited data		
Raw and analyzed data	This paper	GEO:
Experimental models: Cell lines		
Human: HEK293-K^b^	Laboratory of Jonathan Yewdell	N/A
Human: Lenti-X 293T	Takara Bio	Cat# 632180
Experimental models: Organisms/strains		
Mouse: C57BL/6	Jackson Laboratory	N/A
Oligonucleotides		
Oligonucleotide sequences used in this study are provided in Table S1	This paper	N/A
Recombinant DNA		
pcDNA3.1 (myc-His B)-Rps26	This paper	N/A
pRL-CMV-18S rRNA wild type	Burman and Mauro, 2012	N/A
pRL-CMV-18S rRNA mutant	This paper	N/A
Software and algorithms		
GraphPad Prism	Dotmatics	RRID: SCR_002798
Snapgene	Dotmatics	RRID: SCR_015052
R	The R Project	https://www.r-project.org/
Custom Code	This paper	
